# The Effect of Chlorhexidine Mouthwash on Blood Pressure: A Systematic Review and Meta‐Analysis

**DOI:** 10.1111/idh.70035

**Published:** 2026-03-12

**Authors:** Lars S. J. Toonen, Bregje W. M. Van Swaaij, Melissa L. Vagevuur, Fridus (G. A.) Van der Weijden, Mark F. Timmerman, Dagmar Else Slot

**Affiliations:** ^1^ Department of Periodontology Academic Centre for Dentistry Amsterdam (ACTA), University of Amsterdam and Vrije Universiteit Amsterdam Amsterdam the Netherlands; ^2^ Department of Dental Hygiene Hogeschool Arnhem Nijmegen (HAN), University of Applied Sciences Nijmegen the Netherlands; ^3^ Department of Dentistry, Section Implantology and Periodontology Radboud University Medical Center (Radboud UMC) Nijmegen the Netherlands

**Keywords:** blood pressure, chlorhexidine, diastolic blood pressure, mouthwashes, systolic blood pressure

## Abstract

**Aim:**

This study evaluated the effect of rinsing with a chlorhexidine containing mouthwash (CHX‐MW) on blood pressure (BP), as compared to rinsing with a control MW or no MW, based on (randomised) controlled trials and observational studies in humans. PROSPERO CRD42020222495.

**Materials and Methods:**

The PubMed‐MEDLINE and COCHRANE‐CENTRAL databases were searched to identify eligible studies that had been published as of December 2024. Studies evaluating the effect of CHX‐MW on human BP were included. A descriptive analysis of the selected studies and a meta‐analysis was performed.

**Results:**

Searching identified 100 unique studies; five studies fulfilled all inclusion criteria and could be analysed. The descriptive analysis demonstrated that both systolic blood pressure (SBP) and diastolic blood pressure (DBP) were significantly increased after the use of CHX‐MW in two of the included studies. The meta‐analysis (MA) showed no significant difference of means (DiffM) based on end scores between CHX‐MW and controls for SBP (DiffM = 1.56, P=0.17; 95% CI [−0.67; 3.80]) and DBP (DiffM = −0.10, P=0.91; 95% CI [−1.99; 1.78]).

**Conclusion:**

For patients to whom the use of CHX‐MW has been recommended, there is a moderate level of confidence that any change in BP due to the use of the antiseptic MW is insignificant or very small. The clinical importance of this BP elevation is estimated to be negligible.

## 1 Introduction

It has been widely acknowledged that dental plaque plays an essential role in the development of dental decay and periodontal disease [1, 2, 3]. To minimise the impact of dental plaque, mechanical oral hygiene devices are commonly used for its removal. In addition, various products containing chemical ingredients that inhibit plaque formation are available [4]. Antiseptic mouthwashes (MW) containing chlorhexidine (CHX) are commonly used [4] and are currently the preferred antimicrobial agent for chemical plaque control [1, 5]. In dental practice, CHX has been extensively used as an antiseptic since 1970, based on its long‐lasting antibacterial activity and broad‐spectrum efficacy. Numerous clinical trials have demonstrated the effectiveness of CHX in managing dental plaque and reducing gingival inflammation and bleeding. When CHX is dissolved in water, it carries a positive charge, allowing it to attach to the negative cell membranes of bacteria. This attachment leads to changes in the permeability of the cell membrane, disrupting the bacterial osmotic balance. This effect causes leakiness of the cell membrane and ultimately results in cell death [6]. CHX also inhibits bacterial interaction by binding to bacteria, and it prevents plaque colonisation by binding to glycoproteins in saliva.

The use of CHX‐MW has been shown to inhibit the conversion of nitrate to nitrite in the oral cavity, a process that plays a vital role in maintaining vascular health. This disruption has been associated in some studies with increased blood pressure (BP) in normotensive individuals [7, 8]. Oral facultative anaerobic bacteria, which possess nitrate reductase enzymes, are central to this pathway, converting dietary nitrate to nitrite in the oral cavity or stomach [9]. Nitrite, upon swallowing, stimulates nitric oxide signalling, a key mechanism in BP regulation [10]. However, the use of CHX‐MW may disrupt these beneficial oral commensal bacteria, potentially impairing this physiological pathway and its associated health benefits. Nitric oxide is a small, gaseous signalling molecule with multiple functions that contribute to the maintenance of metabolic and cardiovascular homeostasis [11, 12]. Modifying the oral microbiome may potentially result in reduced bioavailability of nitric oxide and hypothetically pose a risk for complications [13]. The mechanism of functioning is as follows: Foods such as beets, lettuce, and spinach are rich in nitrate (NO_3_^−^) [14]. Ingested dietary nitrate is rapidly absorbed in the upper gastrointestinal tract [15]; once in the bloodstream, it is actively taken up by the salivary glands and subsequently secreted in saliva [16]. For nitrate to exert its physiological effects, it must be reduced to the more bioactive nitrite anion. This reduction primarily occurs through the activity of oral commensal bacteria [17]. Various pathways in blood and tissues catalyse the reduction of nitrite to nitric oxide and other nitrogen oxides [11, 12]. It is important to note that the relationship between nitric oxide and vasodilatation typically involves L‐arginine metabolism [18]; bacterial involvement has been less thoroughly investigated [19]. Insufficient production of nitric oxide can contribute to the development and progression of cardiovascular diseases, including hypertension, as measured by systolic blood pressure (SBP) and diastolic blood pressure (DBP) [20]. Consequently, modifying the oral microbiome with an antiseptic MW may impact the regulation of BP through disruption of the nitrite pathway [7].

Most studies investigating the antimicrobial activity of CHX have been conducted in vitro, limiting the investigation of all micro‐organisms present in the oral environment. Although CHX is readily available over the counter and can be used by a part of the general population. It remains unknown whether CHX promotes a healthy oral microbiome or potentially induces a shift towards a microbiome associated with disease. When nitrite formation in the oral cavity is disrupted, it cannot reach the acidic environment of the stomach, impairing the S‐nitrosylation process and its associated physiological effects [21, 22]. S‐nitrosothiols, vasoactive endogenous compounds, are produced in the stomach through the conversion of nitrite. This process is presumed to contribute to BP‐lowering effects observed with both dietary nitrite and nitrate [21, 22].

Due to a lack of extensive research regarding the correlation between nitrate‐reducing bacteria and hypertension [23], there has been an ongoing debate as to whether the use of CHX‐MW clinically results in elevated SBP or DBP, as CHX‐MW can possibly interfere with nitric oxide‐producing bacteria [7, 24, 25, 26]. The absence of a clear pathogenic mechanism has led to controversy and hesitance in completely abandoning or restricting the use of antiseptic MW [13]. Therefore, the purpose of this systematic literature review was to synthesise the existing scientific evidence and assess whether the use of CHX‐MW has an impact on human BP.

## 2 Materials and Methods

This paper was prepared and reported in accordance with the *Cochrane Handbook for Systematic Reviews of Interventions* [27]. In addition, the guidelines of Preferred Reporting Items for Systematic Reviews and Meta‐Analyses (PRISMA) [28] were used. The protocol was developed a priori following an initial discussion among the members of the research team (PROSPERO CRD42020222495) [29]. The Institutional Review Board of the Academic Centre for Dentistry Amsterdam (ACTA) approved this study under protocol number 2023‐58314.

### 2.1 Focused PICOS Question

Based on (randomised) controlled trials and observational studies in humans, what is the effect of rinsing with a CHX‐MW on blood pressure (BP), as compared to rinsing with a control MW or no MW?

### 2.2 Search Strategy

To identify studies for this review, a detailed search strategy was developed and tailored for each database. The electronic databases that were searched included the National Library of Medicine, Washington, DC (MEDLINE‐PubMed), and the Cochrane Central Register of Controlled Trials (CENTRAL). The search covered the period from the inception of each database up to December 2024, aiming to identify suitable studies that had addressed the specific question of interest.

The search terms used for each database are listed in Table 1. These terms were adjusted accordingly for each database, considering variations in controlled vocabulary and syntax rules. There were no restrictions on language or publication date, and any relevant non‐English‐language studies were translated whenever required. No search filters were applied other than trials ‘in humans’, where available.

**TABLE 1:** Search terms used for PubMed‐MEDLINE. The search strategy was customised according to the database being searched. The following strategy was used in the search: [<intervention>] AND [<outcome>].

[< intervention: CHX MW >] (((((((“Mouthwashes”[Mesh]) OR (Mouthwashes OR Mouthwash OR mouthwash* OR mouthrinses OR mouthrinse))))) AND (((“Chlorhexidine”[Mesh]) OR (chlorhexidine OR chlorhexidine di‐gluconate OR (chlorhexidine gluconate) OR zinc‐chlorhexidine OR (chlorhexidine gluconate lidocaine hydrochloride) OR CHX OR (chlorhexidine phosphanilate) OR (CHX formulations) OR (chlorhexidine di‐acetate)))))) AND [< Outcome: BP >] ((((((hypotension OR hypotens*)) OR hypotension [MeSH])) OR (((hypertension OR hypertens*)) OR hypertension [MeSH])) OR ((((blood pressure) OR (systolic pressure) OR (diastolic pressure))) OR blood pressure [MeSH]))

*Note:* An asterisk (*) was used as a truncation symbol.

### 2.3 Screening and Selection

Duplicate references from various databases were identified, and a final set of unique references was created using Rayyan, a web and mobile application for systematic reviews. Rayyan is recommended for its ease of use and suitability in supporting screening for healthcare research [30]. In this review, titles and abstracts were carefully examined to determine their appropriateness. Two reviewers (L.S.J.T. and M.L.V.) independently assessed studies that potentially met the inclusion criteria; this selection process was performed blindly, and studies were categorised as either included or excluded or were marked as undecided.

After this screening process, the search was unblinded, and the conflicts that had been identified by Rayyan [30] were resolved by discussion and consensus; if a disagreement persisted, arbitration by a third reviewer (B.V.S.) was performed. After the list of included titles and abstracts had been obtained, the full‐text publications were retrieved and screened for suitability. At this stage, the reasons for exclusion were recorded. Studies that fulfilled all inclusion criteria were processed for data extraction. The reference lists of the included studies were hand‐searched to identify additional studies that were potentially relevant. If the full text was not retrievable, the paper was excluded.

The inclusion criteria were as follows:

- Randomised controlled clinical trials (RCTs), controlled clinical trials (CCTs), or observational studies
- Conducted on human participants who met the following criteria:
  – Aged ≥ 18 years
  – Normotensive (blood pressure of 130/< 85 mmHg) [31].
  – In good general health (not institutionalised, no systematic disorders)
- Intervention: CHX‐MW (without dietary nitrate supplementation)
- Control: no MW or negative control MW (Studies that lacked a control group, but in which participants were their own control, could also be included)
- Rinsing regimen: [32]
  ○ Daily rinsing
  ○ Follow‐up > 1 day
- Outcome: Blood pressure (DBP and SBP, measured in mm Hg)

### 2.4 Data Extraction

Studies that fulfilled all the inclusion criteria were processed for data extraction using a specially designed and standardised data extraction sheet. The characteristics of the populations, interventions, comparisons, and outcomes were extracted from all studies by two independent reviewers (L.S.J.T. and M.L.V.) for assessment of study quality and synthesis of evidence. Disagreements between the reviewers were resolved through discussion and consensus. If this process was not satisfactory, the judgement of a third reviewer (B.V.S.) was considered decisive. When data were missing, the authors calculated them from the available data or otherwise attempted to obtain them by contacting the authors of the selected papers, if possible. If the standard deviation had not been provided but the standard error was available, the authors calculated the SD based on the sample size (SE = SD/√*N*) [23].

### 2.5 Assessment of Heterogeneity

The factors used to assess the clinical heterogeneity of the outcomes of the various studies were as follows: participant characteristics, duration of the study, concentration of CHX, rinsing regimen, and procedure for BP measurement. When individual studies were sufficiently similar with respect to included patients, treatments, and outcomes, pooling of results was considered and statistical heterogeneity was assessed.

### 2.6 Risk of Bias (Quality) Assessment

All included studies were independently scored for their methodological quality by two reviewers (L.S.J.T. and M.L.V.) using the checklist presented in Appendix S1 in the Supporting Information. Quality criteria were designated with a plus sign (+) if an informative description was present and the study design met the methodological criteria and a minus sign (−) if an informative description was present, but the study design did not meet the criteria. This method has been described in detail by Van der Weijden et al. [33] and Keukenmeester et al. [34], including elements proposed by the *Cochrane Handbook for Systematic Reviews of Interventions* [35]. The list of 20 items was assessed; if all relevant individual items had received a positive rating, an overall score of 100% was assigned [36]. The estimated risk of bias was interpreted as follows: 0%–40% may represent a high risk of bias, 40%–60% may represent a substantial risk of bias, 60%–80% may represent a moderate risk of bias, and 80%–100% may represent a low risk of bias [33, 37].

### 2.7 Strategy for Data Synthesis

Data from the included studies were categorised according to either SBP or DBP. As a summary, descriptive data presentation was employed [38]. In addition, when feasible, a meta‐analysis (MA) was performed (only if two or more studies could be included) [39].

### 2.8 Pooling of Data

Meta‐analyses were performed using the Review Manager (RevMan) programme [40], version 5.4, in accordance with the COCHRANE handbook [27] and the PRISMA guidelines [28]. Comparisons of interest were CHX‐MW versus a control MW with respect to end scores and differences between baseline and end scores for incremental data. For studies using an observational design, the baselines or the scores after placebo use served as controls. Pooled outcome was expressed as difference of means (DiffM) with its associated 95% confidence interval (CI). DiffM was calculated using an inverse variance method with a fixed effects or random effects model where appropriate. The random effects model was generally used, as this model is well suited for meta‐analysis with heterogeneous effects. A fixed effects model was applied for cases in which fewer than four comparisons had been performed, as the estimate of between‐study variance is poor for analyses with low numbers of studies. A *p*‐value below 0.05 was considered to indicate significance.

Heterogeneity was tested using the chi‐squared test and the *I* ^2^ statistic [27]. A chi‐squared test resulting in a *p*‐value below 0.1 was considered to be an indication of significant statistical heterogeneity. As an approximate guide for assessing the degree of inconsistency across studies, an *I* ^2^ statistic of 0%–30% was interpreted as potentially not important; a value of 30%–60% was taken to represent moderate heterogeneity, and 60%–90% was taken to represent substantial heterogeneity [27].

### 2.9 Publication Bias

If the meta‐analysis had considered sufficient trials to make visual inspection of the plot meaningful (a minimum of 10 trials), funnel plots were used for assessment of publication bias as proposed by Egger et al. [41]. Presence of asymmetry in the inverted funnel was considered to suggest publication bias [27].

### 2.10 Grading the ‘Body of Evidence’

In order to present an overall clinical recommendation based on the outcome of the present systematic review, a modified version of aspects regarding the Grading of Recommendations Assessment, Development and Evaluation (GRADE) [42] was used. Two authors (L.S.J.T. and D.E.S.) rated the quality of the evidence and the strength and direction of the recommendations according to the following aspects: risk of bias, consistency of results, directness of evidence, precision of the data, presence of publication bias, and magnitude of the effect. In addition, the overall direction and strength of the confidence in effect estimates for the main outcome was rated. At last, based on these aspects together as summary of findings an overall clinical recommendation was made. Any disagreement between the two reviewers was resolved through additional discussion with a third author (B.V.S.).

## 3 Results

### 3.1 Search and Selection Results

**FIGURE 1:**
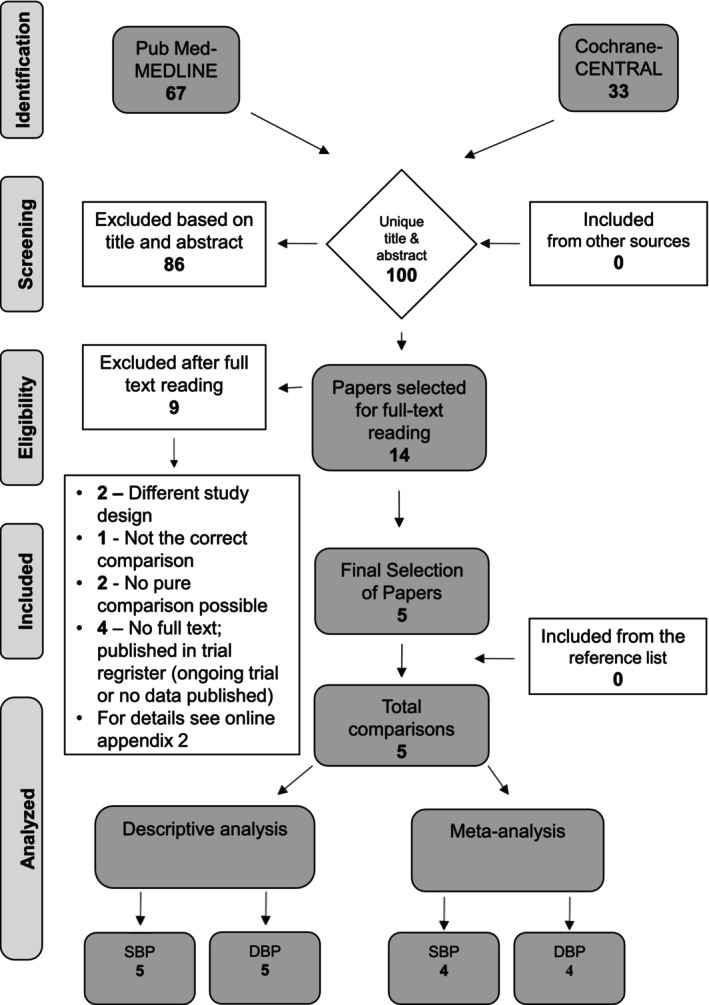
Flow chart: Search and selection results with reasons for exclusion.

Searching MEDLINE‐PubMed and Cochrane‐CENTRAL databases identified 100 unique studies (Figure 1). All studies were independently screened, and a selection was performed by the reviewers with an overlap of 100%. In total, four studies (Kapil et al., 2013 [I]; Sundqvist, Lundberg & Weitzberg, 2016 [II]; Tribble et al., 2019 [III]; and Bescos et al., 2020 [IV]) [8, 11, 24, 26], fulfilled all inclusion criteria and were processed for data extraction. Bescos et al., 2024 [V] [43] was included although it has a positive control group. However, only the baseline and END scores of the CHX group were used for further analysis. After full‐text reading, nine studies were excluded (Appendix 2 in the Supporting Information). A flow chart in Figure 1 summarises the study selection process.

### 3.2 Assessment of Heterogeneity

The five selected studies exhibited heterogeneity in study design, participant characteristics, study duration, duration of rinsing, CHX concentration, CHX‐MW rinsing regimen, method, and procedure for BP measurements. Table 2 presents further details.

**TABLE 2:** Overview of details regarding the studies processed for data extraction.

Study number, authors, year, country, risk of bias	Study design, duration	Participant base (end), gender, age (mean, range)	Method of BP measurement, regimen, brand of CHX	Conclusions of the original authors
I—Kapil et al. 2013 United Kingdom RoB: moderate	Observational study Double‐blind NCG Intervention: 7 days Washout: —	19 (19) ♀: ? ♂: ? Mean age: 23.8 Age range: 18–45 General health: no systemic medication; nonsmokers; no antibiotic use; no history of or recent treatment for an oral condition (excluding caries), including gingivitis, periodontitis, and halitosis	BP: semi‐automated oscillometric machine Omron 715IT (Omron Corp., Tokyo, Japan) every 15 min for 1 h MW: CHX‐MW 10 mL Corsodyl (0.2% CHX, GlaxoSmithKline, UK) twice daily. Per rinse: 20 mg CHX ◊	Seven days' use of antiseptic MW caused a rise in clinical SBP and DBP. The BP effect appeared within 1 day and was sustained during the 7‐day MW intervention.
II—Sundqvist et al. 2016 Sweden RoB: moderate	RCT Randomised, double blind, crossover Intervention: 3 days Washout: 28 days	19 (17) ♀: 17 ♂: 0 Mean age: 23 Age range: 19–27 General health: No chronic medication, no antibiotic use, nonsmokers	BP: Omron M10‐IT (Omron Corporation, Japan) three times; the second and third reading were averaged to determine a mean clinical BP. MW: CHX‐MW (0.2% CHX) rinsed 3 times per day, 1 min each time. per rinse: ? mg CHX MW placebo without CHX	In young healthy females, use of an antiseptic MW (with CHX) was not associated with changes in BP.
III—Tribble et al. 2019 United States of America RoB: substantial	Observational study NCG Intervention: 7 days Washout: —	28 (26) ♀: 17 ♂: 10 Mean age: 31.8 Age range: 22–71 General health: nonsmokers, no antibiotic use, no history of bone loss (periodontitis), no history of hypertension or current pregnancy	BP: Omron 10 BP was taken on alternating arms at each time point, beginning with the right arm. MW: CHX‐MW Peridex (0.12% CHX‐gluconate) twice per day rinse with ½ ounce (±15 mL) for 30 s per rinse: 18 mg CHX ◊	Twice‐daily CHX use was associated with a significant increase in SBP after 1 week.
IV—Bescos et al. 2020 United Kingdom RoB: substantial	Observational study Single‐blind NCG Intervention: 7 days Washout: —	36 (36) ♀: 11 ♂: 25 Mean age: 26 Age range: ? General health: nonsmokers, not suffering from gingivitis or periodontitis or exhibiting a medical condition (e.g., hypertension, diabetes)	BP: Connex ProBP 3400 Digital Blood Pressure Device, Welch Allyn UK Ltd. Participants rested in a supine position for 30 min; three readings were taken (four if variation in SBP or DBP of > 4 mmHg was found) with 1 min rest between readings. The second and third readings were averaged to determine mean clinical BP. MW: CHX‐MW Corsodyl Mint (0.2% CHX) (GlaxoSmithKline, UK) 10 mL for 1 min, twice per day. per rinse: 20 mg CHX ◊	Use of CHX was followed by a trend of increased systolic blood pressure.
V—Bescos et al. 2024 United Kingdom RoB: moderate	RCT Randomised, double blind. Intervention: 7 days Washout: —	24 (21) ♀: 17 ♂: 4 Mean age: 26 Age range: 18–50 General health: Healthy adults with a BMI < 30 kg/m and no following periodontal treatments or using antimicrobial products and antibiotics within 3 months before initiation of study. Participants with a periodontal examination score of 2 or lower, but no more than one BPE 2 in any sextant were included in this study.	BP: BP was measured in triplicate using an electronic BP monitor (Connex ProBP 3400, Welch Allyn UK) after resting for 10 min. The second and third readings were averaged to determine mean BP. MW: CHX‐MW Corsodyl (0.2% CHX), GlaxoSmithKline, UK 10 mL, duration ?, twice per day. per rinse: 20 mg CHX Propolis‐MW (2.5% propolis), from the Prades mountains, Tarragona, Spain. 10 mL, duration ?, twice per day. per rinse: 0.4 mg propolis extract.	No BP increase in the CHX‐MW group was observed.

Abbreviations: —, no; ?, unknown/not provided; ◊, calculated by the authors of this review based on the presented data in the selected paper; BP, blood pressure; CHX, chlorhexidine; DBP, diastolic blood pressure; MW, mouthwash; NCG, no control group (participants served as their own control); NO, nitric oxide; RCT, randomised controlled trial; RoB, risk of bias score (see Appendix 1 in the Supporting Information for more information); SBP, systolic blood pressure.

#### 3.2.1 Study Design

Of the five included studies, only two (II and V) used an RCT design. Three studies (I, III, IV) used an observational design that had no control group but used baseline data as their own control. Two studies (II, IV) applied a crossover design. In study II, participants first rinsed for a period of time with either a placebo or the CHX‐MW, followed by the opposing sequence. In study IV, participants first rinsed with a placebo before the trial period and then with CHX‐MW. Study durations for the interventions ranged from 3 (IV) to 7 (I, III, IV, V) days.

#### 3.2.2 Participant Characteristics

The number of participants varied from 19 (I) to 36 (IV) participants. The age of participants ranged from 18 to 71 years, and one study (II) focused on young adults between 19 and 27 years of age. Three studies (III, IV, V) analysed a mixed‐gender population, while one study (II) only selected female participants. In all studies, the participants were non‐smokers. Exclusion criteria were the presence of known hypertension (III, IV), pregnancy (II), presence of a medical condition such as diabetes (IV), BMI < 30 kg/m (V), and regular or active attempts to lose weight (II). Papers I and II specifically mention that no systemic medication had been allowed except birth control pills. In addition, participants had been excluded if they had recently been prescribed antibiotics (I, II, III, V).

#### 3.2.3 CHX‐MW

Variability was noticed in the concentrations and instructions for use of the CHX‐MW products. Four studies used 0.2% CHX, mostly Corsodyl (I, II, IV, V), and one study (III) used 0.12% CHX (Peridex). The amount of MW used for rinsing ranged from 10 mL (I, IV, V) to approximately 15 mL (III). Rinsing events ranged from 2 (I, III, IV, V) to 3 (II) times daily, and the rinsing time varied from 30 s (III) to 1 min (II, IV).

#### 3.2.4 BP

Methods for measuring BP differed with respect to measuring device and procedure. In all studies, an automatic system was used. Three studies used an Omron device (I, II, III), and two used a Connex (IV, V). Table 2 presents details regarding the procedures used to measure BP.

#### 3.2.5 Dietary Restrictions

In studies I and II, subjects received dietary restrictions, including intake of low‐nitrate vegetables, and fasted before all appointments. No details regarding diet were provided for study III. In study IV, participants had fasted overnight and had been reminded to avoid drinking caffeine products and to refrain from strenuous exercise. Participants in study V attended the laboratory under fasting conditions (> 3 h).

#### 3.2.6 Conflict of Interest and Funding

All five included studies (I, II, III, IV and V) received funding from a foundation, trust, university, or institution. Two authors of paper II had been listed as co‐inventors on patent applications related to the therapeutic use of inorganic nitrate. Paper III describes in detail the competing interests of the different authors.

### 3.3 Assessment of Methodological Quality

The potential risk for bias was estimated based on the methodological quality of the selected studies, as presented in Appendix 1 in the Supporting Information. Based on a summary of the proposed criteria for bias assessment, the potential risk of bias was estimated to be ‘substantial’ for studies III and IV and ‘moderate’ for studies I, II and V.

### 3.4 Study Outcome

Appendices 3 and 4 in the Supporting Information present the data extraction that was performed on the selected studies for both SBP (Appendix 3a in the Supporting Information) and DBP (Appendix 3b in the Supporting Information). For observational studies, participants served as their own controls.

#### 3.4.1 Description of Findings

Table 3 presents a descriptive summary of statistical significance levels presented in the selected papers regarding the effect of the use of CHX‐MW on BP as compared to controls. Two studies (I, III) found a significant increase in both SBP and DBP.

**TABLE 3:** A descriptive summary of statistical significance levels regarding the effect of CHX‐MW use on SBP and DBP.

Authors (year)	Intervention	SBP	DBP
(III) Tribble et al. (2019)	CHX‐MW 0.12%	+	+
(I) Kapil et al. (2013)	CHX‐MW 0.2%	+	+
(II) Sundqvist et al. (2016)	CHX‐MW 0.2%	o	o
(IV) Bescos et al. (2020)	CHX‐MW 0.2%	o	o
(V) Bescos et al. (2024)	CHX‐MW 0.2%	o	o

*Note:* 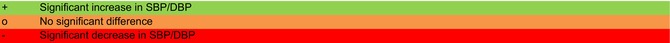

Abbreviations: CHX, chlorhexidine; DBP, diastolic blood pressure; MW, mouthwash; SBP, systolic blood pressure.

#### 3.4.2 Meta‐Analysis

**FIGURE 2:**
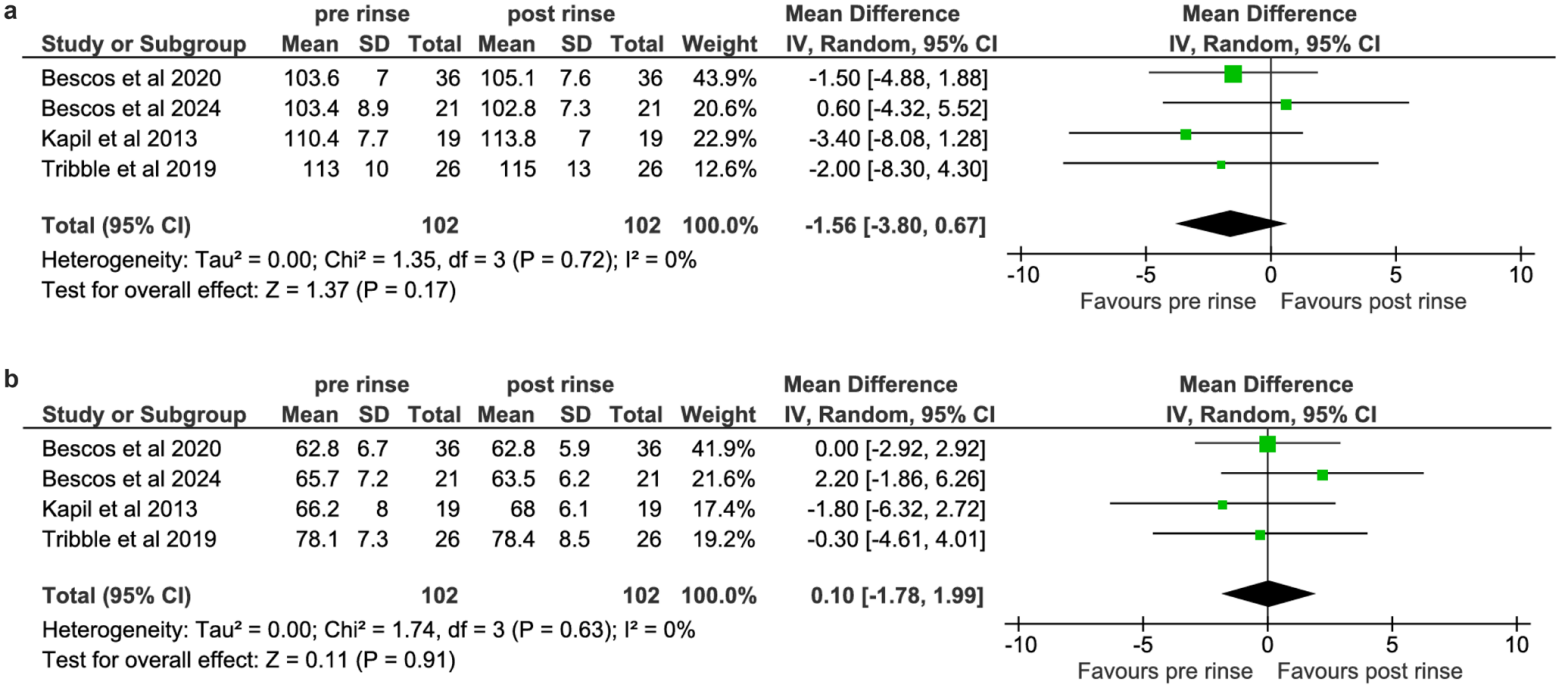
(a) Meta‐analysis evaluating the effect of CHX mouthwash use on SBP, comparing pre‐rinse versus post‐rinse measurements. (b) Meta‐analysis evaluating the effect of CHX mouthwash use on DBP, comparing pre‐rinse versus post‐rinse measurements.

A meta‐analysis could be performed on data emerging from observational studies. The meta‐analysis is shown in Figure 2a,b for SBP and DBP, respectively. The meta‐analysis of post‐rinsing scores as compared to the control data showed no significant difference of means for SBP (DiffM = 1.56, P=0.17; 95% CI [−0.67; 3.80]) or DBP (DiffM = −0.10, P=0.91; 95% CI [−1.99; 1.78]). No statistical heterogeneity (*I* ^2^ = 0%) was observed; therefore, heterogeneity was interpreted as potentially not important.

### 3.5 Evidence Profile

Table 4 presents a summary of the various aspects by which the quality of the evidence was rated and an appraisal of the strength and direction of the effect. Altogether, the recommendation to those patients who have been advised to use CHX‐MW is that there is a moderate level of confidence that any potential increase in BP is likely to be minor or negligible.

**TABLE 4:** Summary of findings based on the quality and body of evidence from the estimated evidence profile [42] and appraisal of the strength of the recommendation regarding the effect of rinsing with a CHX‐MW on BP.

Study outcome	SBP	DBP
# experiments in descriptive analysis (Table 3)	5	5
# experiments in meta‐analysis (Figure 2a,b)	4	4
Risk of bias (Appendix 1 in the Supporting Information)	Low–high	Low–high
Consistency	Rather inconsistent	Rather inconsistent
Directness	Rather generalisable	Rather generalisable
Precision	Rather precise	Rather precise
Reporting bias	Possible	Possible
Magnitude of the effect	None	None
Strength and direction of the recommendation	Moderate	Moderate
Overall recommendation	**For normotensive patients advised to use CHX‐MW, there is a moderate level of confidence that any potential increase in blood pressure due to the use of the antiseptic mouthwash is likely to be minor or negligible.**	

## 4 Discussion

### 4.1 Summary of Key Findings

The primary aim of this review was to systematically synthesise data regarding the effect of rinsing with CHX‐MW on BP, as described in the current literature. Most included papers were published in non‐dental journals. In the descriptive analysis, two of the five included comparisons detected a significant increase in SBP and DBP after the use of CHX‐MW. Meta‐analyses of four studies (I, II, IV, V) found no significant change in SBP and DBP before or after rinsing. Small, non‐significant DiffM values of 1.56 mmHg for SBP and −0.10 mmHg for DBP were found.

### 4.2 Nitric Oxide

In general, the use of CHX‐based antiseptic MW has demonstrated a reduction of the conversion of nitrate to nitrite by oral bacteria. This effect has been associated in some studies with an increase in BP in normotensive subjects; this finding has also emerged from the present review [44]. However, while twice‐daily CHX usage was associated with a significant increase in systolic BP after 1 week of use, recovery after use resulted in an enrichment of nitrate‐reducing bacteria on the tongue. Individuals bearing relatively high levels of bacterial nitrite reductases had lower resting SBP, indicating that high levels of bacterial nitrite reductases may provide an opportunity for the improvement of resting SBP [24]. Nitrate and nitrite serve as reservoirs and perform beneficial, nitric‐oxide‐like biological functions. Exogenous dietary nitrate plays an important role in various physiological activities as an effective supplement of nitrite and nitric oxide in the human body. Nitric oxide is essential in multiple physiological processes [9]; it is a potent endogenous vasodilator and also plays an important role in lowering lipid levels, inhibiting the expression of adhesion molecules, aggregating platelets, and regulating vascular smooth muscle cell proliferation [45]. Reduction in nitric oxide bioavailability is associated with the occurrence or worsening of pathologies such as atherosclerosis, diabetes, and sepsis [13]. The nitrate–nitrite–nitric oxide pathway is emerging as an important mediator of blood flow [46]. The source of dietary nitrate is mainly green, leafy vegetables, meat, and drinking water, absorbed into the blood through intestinal mucosa. After consuming a meal rich in nitrates, plasma nitrate is concentrated in saliva by the salivary glands, resulting in high nitrate concentrations that can reach the millimolar range [47]. Human cells cannot reduce nitrate to nitrite effectively. Facultative anaerobic bacteria in the oral environment are critical contributors to the nitrate–nitrite–nitric oxide pathway by converting nitrate into nitrite, either in the mouth or the stomach [9]. Nitrite is subsequently converted to nitric oxide through non‐enzymatic synthesis [9]. Nitrite in stimulated saliva has been shown to correlate with plasma nitrite levels [48]. Salivary levels in the oral cavity are 1000‐fold higher than those in plasma [49]. The continuous swallowing of nitrite‐containing saliva (~1.5 L per day) generates a variety of nitrogen oxides, including nitric oxide, in the acidic milieu of the gastric lumen.

Nitrite also correlates with periodontal status, showing increased levels in individuals with periodontal disease. During the development of periodontal disease, human gingival fibroblasts produce nitric oxide in response to proinflammatory cytokines [50]. Four of the papers (I, III, IV, V) included in the present review report limited details regarding the periodontal health status of the included participants, and one paper does not provide any specific information (II). The four papers (I, III, IV, V) that did provide information on periodontal status only included participants with a healthy periodontal status; see Table 2 for more information. In future research, it would be interesting to analyse the effect of CHX in patients with the actual indication for the use of this specific rinse. A 6‐month randomised clinical trial was conducted based on the presumption that periodontal treatment would be followed by a reduction of nitrite levels in saliva, which could be related to oral microbial improvements. The results showed that after periodontal therapy, the relationship between nitrite and bacterial levels was weak and that full mouth scaling within 24 h exhibited a greater influence on nitrite concentrations than long‐term (60‐day) CHX use [51].

Nitrite concentration in the human body may also have side effects, including the formation of nitrate metabolites such as N‐nitrosamines. While consumption of a diet rich in nitrate and amines increases the risk of formation of carcinogenic nitrosamines, the use of an antiseptic MW containing CHX can also have positive effects that result in inhibition of nitrosamine formation [52, 53]. The nitrate–nitrite–nitric oxide pathway can also affect the composition and metabolism of the oral microbiome. The addition of nitrate to oral communities has been shown to lead to rapid modulation of microbiome composition and activity that could be beneficial for the host [54]. It has been proposed that nitrate should thus be investigated as a potential probiotic for oral health. It is suggested that oral hygiene measures are likely to favour denitrifying bacteria [55]. Based on this observation, a model has been proposed to link lifestyle factors with oral and systemic health through nitric oxide metabolism [55]. It remains to be clarified whether the use of CHX‐MW has an effect on the enterosalivary cycle as a result of a decreased or altered oral microbiome [23]. Two studies were not considered in the present systematic review because they met all but one inclusion criterion [7, 25]. Nitrate concentration was optimised, as drinking beetroot juice had been added as part of the study regimen, which could potentially influence or bias the effect compared to a normal or low nitrate diet.

### 4.3 Measuring Blood Pressure

BP measurement is a routine procedure conducted in various settings such as primary care, healthcare facilities, pharmacies, and even at home. A sphygmomanometer is commonly used to indirectly measure arterial BP. It is important to note that the displayed values may not always reflect the true BP [56]. BP serves as an essential vital sign and plays a pivotal role in diagnosing hypertension. Accurate measurement of BP is a fundamental element in evaluating various medical conditions and ensuring the reliable diagnosis and effective treatment of hypertension [57, 58]. A systematic review examined 29 potential sources of inaccuracy, categorised into patient‐related, device‐related, procedure‐related, or observer‐related factors [59]. These authors have advised that a single BP value outside the expected range should be interpreted with caution and should not be considered a definitive indicator of clinical deterioration. In the studies included in this systematic review, multiple readings were taken and a mean value was calculated. Conducting more comprehensive resting BP measurements could potentially reduce the variability of the recorded values [26].

With the exception of one report (I), the studies included in the present systematic review used automated systems for BP measurements (Table 2 for details). It has been demonstrated that automated devices are generally less accurate than manual assessment methods [60]. However, manual assessments require extensive training for examiners [60]. The report of study I, which applied a semi‐automatic BP measurement method, includes only an instruction on the correct use of the device. Research has shown that manual BP measurements are generally higher than measurements obtained with automatic devices [61] as also illustrated in the forest plots presented in Figure 2. Nonetheless, because the confidence intervals overlap with those of the other studies, this difference does not appear to have influenced the overall findings significantly. Most automated BP devices are calibrated, and some have the option of self‐calibration. In most cases, automatic devices appear to be sufficiently accurate for clinical use [60]. Regulatory authorities worldwide enforce independent validation before granting approval for marketing of devices that measure BP [57]. According to the American National Standards Institute, Association for the Advancement of Medical Instrumentation and International Organisation for Standardisation protocol, the reported accuracy of Omron devices for BP measurement is ±3 mmHg [62], and it has been reported to be even higher [63]. This observation indicates that the detected differences (DiffM) in this meta‐analysis (ranging from −0.6 to 3.5 for SBP and −2.2 to 2.2 for DBP) are near the margin of measurement error. Therefore, caution should be exercised when interpreting the effect size as derived from this review.

### 4.4 Blood Pressure Value Interpretation

Based on the 2020 International Society of Hypertension Global Hypertension practice guidelines, which classify ‘normal’ BP as < 130/< 85 mmHg, the baseline data presented in Appendices 2a and 2b in the Supporting Information demonstrate that the mean SBP and DBP levels of the included participants align well with the normal range [31]. These mean values also fall below the optimal BP level recommended by the Heart Foundation (120/80 mmHg) [64]. Considering the values for normal BP even after the use of CHX, the incremental increases observed (ranging from −0.6 to 3.5 for SBP and −2.2 to 2.2 for DBP) did not result in BP values that exceeded the normal range. Although two studies (I, III) showed a statistically significant increase, the magnitude of this increase appears to have been ‘clinically insignificant’. Clinically important changes have been defined as increases in systolic BP greater than 20 mmHg and increases in DBP greater than 10 mmHg [65]. All eligible studies for the present review included normotensive participants. The data that emerged are also in line with a study that assessed the effects of 3 days of CHX‐MW use in a treated hypertensive population [66]. This study found in normotensive BP patients that the use of CHX‐MW resulted in a small elevation of 1.56 mmHg in SBP. Therefore, healthcare professionals, particularly those involved in dental care, may confidently prescribe CHX‐MW for those patients when indicated, considering that the potential effect on BP lies safely within acceptable limits.

### 4.5 Statistical Power

In the present review, two studies (I, III) reported a significant effect on both DBP and SBP following the use of CHX. Notably, these two studies are the only ones that performed an ‘a priori’ sample size calculation. On the other hand, the remaining studies, which did not perform these calculations and did not show a significant effect, may have suffered from insufficient statistical power. However, it is worth noting that underpowered studies tend to contribute little information when examined in Cochrane reviews [67] and that the advantage of conducting a meta‐analysis is that it allows the combination of individual studies to provide a single and more precise estimate of treatment effects [68]. The meta‐analysis conducted in the present study detected potentially non‐important and non‐significant statistical heterogeneity. The *I* ^2^ value of 0% indicates that the observed variance likely reflects real differences in effect size [69].

### 4.6 Measurement Bias

Many individuals often experience some level of anxiety when visiting a medical office, which can lead to elevated BP readings. This phenomenon, known as ‘white‐coat hypertension’, causes temporary increases in BP during medical appointments. It has been suggested that approximately 20% of patients suffer from this effect [70]. It has been further suggested that measurements in a nonclinical setting by trained technicians can avoid the ‘white‐coat’ bias and therefore improve diagnosis [71, 72]. In study II, participants were placed in a quiet environment for 15 min and two manual BP readings were taken. In study III, the measurements were taken in a dental chair in an upright position (III), in study IV, participants rested in a supine position for 30 min before three successive readings were taken and in study V BP readings were taken after resting for 10 min. In study I, both the operator and the volunteer were blind to the measurement outcomes.

### 4.7 Recommendations

Due to the limited details available in the included papers, which had been published in non‐dental journals, regarding the periodontal health status of participants and specific details regarding CHX‐MW use, future research should aim to provide more comprehensive insight into these aspects. Because periodontal health has been shown to influence nitric oxide production [50], understanding its impact on study outcomes is essential.

To mitigate potential bias from ‘white‐coat hypertension’ in BP measurements, it is recommended to employ ambulatory BP measurements. This method involves recording BP continuously for 24 h or longer, with readings taken every 15–30 min throughout the day and night while patients engage in their regular activities [70, 72]. In addition, considering the use of virtual reality (VR) technology to present relaxing scenes, isolating patients from real medical environments, could be a promising alternative to avoid white‐coat hypertension and enhance measurement accuracy [73].

Considering the short durations of the included studies (3–7 days), further investigations should explore the effects of longer‐term CHX‐MW use and assess how long the impact of CHX‐MW persists. This information would offer valuable insights into the sustained effects of CHX‐MW treatment. This review included only studies that investigated the effects of CHX‐based mouthwash in individuals with normotensive BP. Among individuals at risk, such as those who are overweight or obese, frequent use of mouthwash (twice or more daily) has been associated with a higher risk of developing hypertension compared to less frequent use or non‐use [10]. However, as the referenced study does not specify the type of mouthwash or the duration of its use, further research is warranted. Future investigations should focus on assessing the impact of short‐term use of CHX‐MW on BP in specific populations, particularly those with elevated BP or an increased risk of hypertension due to lifestyle‐related diseases, to confirm its safety in these groups.

## 5 Conclusion

For patients with normotensive BP who have been advised to use CHX‐MW, there is a moderate level of confidence that any increase in BP due to the use of the antiseptic MW may be insignificant or very small. The clinical importance of this BP elevation is estimated to be negligible.

## 6 Clinical Relevance

### 6.1 Scientific rationale for the study

Chlorhexidine mouthwash (CHX‐MW) is currently the gold‐standard antimicrobial agent for plaque control. It has been suggested that its effect on the composition of the oral flora may negatively influence nitrite‐related regulation of blood pressure (BP).

### 6.2 Principal findings

The use of CHX‐MW is associated with a very small elevation of BP, at the most. The clinical relevance of this effect is considered to be negligible.

### 6.3 Practical implications

In patients with normotensive BP the use of a CHX‐MW is safe with regard to potential risks of adverse effects on BP elevation.

## Author Contributions

All authors gave final approval and agreed to be accountable for all aspects of work, ensuring integrity and accuracy. L.S.J.T. contributed to design, search and selection, analysis, and interpretation and drafted the manuscript. B.V.S. contributed to conception and design, search and selection, analysis, and interpretation, and critically revised the manuscript. M.L.V. contributed to design, search and selection, analysis, and interpretation, and drafted the manuscript. G.A.W. contributed to conception and design, analysis, and interpretation, and critically revised the manuscript. M.F.T. performed analysis and interpretation and critically revised the manuscript. D.E.S. contributed to conception and design, analysis, and interpretation and critically revised the manuscript.

## Funding

This study was sponsored in part by an unrestricted educational grant from HALEON for the project ‘CHX mouthwash a 360degree Clinical View’ to Slot. The company had no say in the design of this review, nor did it influence the reporting and publishing of the findings. They have approved the content of this manuscript.

## Conflicts of Interest

The authors declare no conflicts of interest. This study was prepared as a part of the obligation of Toonen and Vagevuur to fulfil the requirements of the bachelor's programme in Dental Hygiene, Hogeschool Arnhem Nijmegen (HAN), University of Applied Sciences, Nijmegen, The Netherlands. This research received no other specific grant from any funding agency in the public, commercial, or not‐for‐profit sectors. No funding was accepted for this study other than support from the listed institution, as work for this paper was funded by a regular academic appointment at the Academic Center for Dentistry Amsterdam (ACTA) of Slot and Van der Weijden, HAN University of Applied Sciences of van Swaaij, and Radboud University of Timmerman. Van der Weijden, Slot, and their research team at ACTA have previously received external advisor fees, lecturer fees, or research grants from manufacturers of dental care products. These manufacturers include Colgate, Curaprox, Curasept, Dentaid, GSK, HALEON, Listerine, Oral‐B, Procter & Gamble, and Sunstar.

## Supporting information


**Appendix S1:** idh70035‐sup‐0001‐Supinfo1.pdf.

## Data Availability

Data were derived from public domain resources and, if required, detailed requests to the original authors. Data supporting the findings of this study are available in the Supporting Information for this article.
