# Supplementary material for: The Effect of Chlorhexidine Mouthwash on Blood Pressure: A Systematic Review and Meta‐Analysis
Source: Int J Dent Hyg. 2026 Mar 12;24(3):390–403. doi: 10.1111/idh.70035 (PMC13309218; doi:10.1111/idh.70035)
Supplement: Supplementary file 1 — Appendix S1: idh70035‐sup‐0001‐Supinfo1.pdf. [file IDH-24-390-s001.pdf]

The effect of chlorhexidine mouthwash on blood pressure: A systematic review and meta-analysis

L.S.J. Toonen (https://orcid.org/0000-0001-6619-853X)
B.W.M. van Swaaij (https://orcid.org/0000-0002-7364-8612)
M.L. Vagevuur (https://orcid.org/0000-0002-7364-8612)
G.A. Van der Weijden (https://orcid.org/0000-0002-5075-8384)
M.F. Timmerman (https://orcid.org/0000-0003-1432-6987)
D.E. Slot (https://orcid.org/0000-0001-7234-0037)

Online Appendix

Supporting information
Additional Supporting information may be found in the online version of this article.

Online Appendix 1
Methodological quality and potential risk of bias scores for the individual included studies.

Online Appendix 2
Overview excluded papers after full text reading.

Online Appendix 3a
Mean (SD) scores for the different intervention groups, including various indices and their effects on SBP.
Online Appendix 3b
Mean (SD) scores for the different intervention groups, including various indices and their effects on DBP.

Online Appendix 4
Overview of the included studies and the methodological items applied in the three types of meta-analysis performed.

Online Appendix 5
List of abbreviations

Online Appendix 1. Methodological quality and potential risk of bias scores for the individual included studies.

Study		I<br>Kapil et al. (2013)	II<br>Sundqvist et al. (2016)	III<br>Tribble et al. (2019)	IV<br>Bescos et al. (2020)	V<br>Bescos et al. (2024)
**Internal validity**	1. Study design	Observational study	RCT	Observational study	Observational study	RCT
	2. Random allocation	–	+	–	–	+
	3. Allocation concealment	–	+	NA	NR	NR
	4. Blinded to product	–	+	–	NR	+
	5. Blinded to examiner	–	+	–	NR	+
	6. Blinding during statistical analysis	NR	NR	NR	NR	NR
	7. Balanced experimental groups	+	+	+	+	+
	8. Reported loss to follow-up	+	+	+	+	+

	# (%) of dropouts	0 (0%)	2 (10.5%)	2 (7.1%)	0 (0%)	3 (12,5%)
	9. Treatment identical, except for intervention	+	+	+	+	+
**External validity**	10. Representative population group	+	+/–	+	+	+/-
	11. Eligibility criteria defined	+	+	+	+	+
	12. Sample size calculation and power	+	–	+	–	+
	13. Point estimates presented for the primary outcome mean	+	–	+	–	+
	14. Measures of variability presented for the primary outcome SD/CI	+	–	–	–	-
	15. Unit of analysis	subject	subjects	subjects	subjects	subjects
	16. Included a per-protocol analysis	NR	NR	NR	NR	NR
	17. Included an intention-to-treat analysis	NR	NR	NR	NR	NR

**Clinical aspects**	18. Validated measurement	+	+	NR	+	+
	19. Calibration examiner	+	+	+	+	+
	20. Reproducibility data shown	+	+	–	–	-
**Authors' estimated quality score**		65%	67.5%	50%	45%	67,5%
**Authors' estimated risk of bias**		moderate	moderate	substantial	substantial	moderate

Each aspect of the reporting and methodological quality item score list was assigned a rating of plus ("+") for informative description of the item at issue and meeting the quality standard; plus–minus ("+/–") was assigned if the item was incompletely described, and minus ("–") was used if the item was not described at all (Hidding et al., 2014).[25] NA = not applicable, and NR = not reported/unclear. For the quality assessment score, individual items with a positive rating were summed to obtain an overall percentage score.[28]

Oline Appendix 2. Excluded papers after full text reading.

Author, Year	Reason for rejection
Cochrane library (2023-2024)	Title: The Effect of Chlorhexidine on the Oral Microbiome and Saliva in Dental Erosion – No full text available; Ongoing study
Cochrane library (2023)	Title: The Effect of Nitrate Supplement on the Oral Microbiome and Saliva in Dental Erosion – No full text available; Ongoing study
Cochrane library (2021)	Title: TO STUDY THE EFFECT OF CHLORHEXIDINE MOUTHRINSE ON BLOOD PRESSURE, NITRATE AND NITRITE LEVELS (SERUM AND SALIVARY) AFTER SCALING IN PATIENTS with severe gum infection – No full text available; No data posted, no results available, no publication according the Cochrane library
Cochrane library (2021)	Title: EFFECT Of nitrate metabolism difference in volunteers using herbal mouthwash and those using chlorhexidine mouthwash - No full text available; Study has not started
Pignatelli et al. 2020	Different study design
Preshaw et al. 2018	Different study design
Woessner et al. 2016	(dietary nitrate supplementation) incorporated into discussion
McDonagh et al. 2015	(dietary nitrate supplementation) incorporated into discussion
Cortelli et al. 2015	No comparison in blood pressure

Online Appendix 3a. Mean (SD) scores for the different intervention groups, including various indices and their effects on SBP.

**Study**	**Measurement device**	**Group**	**Baseline (mm Hg)**	**End (mm Hg)**	**Difference (mm Hg)**
I Kapil et al. (2013)#	IClinic BP Omron 715IT (Omron Corp., Tokyo, Japan)	CHX-MW 0.2%	110.4 (7.7)♦	113.8 (7.0)♦	3.5 (4.4)♦
II Sundqvist et al. (2016)	Omron M10-IT	CHX-MW 0.2%	NA♦	110.0 (6.1)♦	NA♦
		Placebo	NA♦	109.2 (5.8)♦	NA♦
III Tribble et al. (2019)#	Omron 10	CHX-MW 0.12%	113.0 (10)♦	115.0 (13)♦	#2.0 (?)◊
IV Bescos et al. (2020)^	Connex ProBP 3400 Digital Blood Pressure Device, Welch Allyn UK Ltd.	CHX-MW 0.2%	103.6 (7.0) ♦	105.1 (7.6)♦	1.5 (4.3)♦
		Placebo	NA♦	103.6 (7.0)♦	NA♦
V Bescos et al. (2024)	Connex ProBP 3400 Blood Pressure Device, Welch Allyn UK Ltd.	CHX-MW 0.2%	103.4 (8.9)♦	102.8 (7.3)♦	-0.6 (4.9)♦
		Control	102.2 (7.0)♦	100.2 (7.9)♦	-2.0 (4.2)♦

? Unknown/not provided
◊ Calculated by the authors of this review based on the data presented in the selected paper
♦ Provided by the original author
NA Not applicable
No control group (only a CHX-MW group); participants served as their own controls.
^ Observational crossover design; end scores after placebo use served as baseline for the CHX measurement, and participants served as their own controls.

Online Appendix 3b. Mean (SD) scores for the different intervention groups, including various indices and their effects on DBP.

Study	Measurement device	Group	Baseline mm Hg	End mm Hg	Difference mm Hg
I Kapil et al. (2013)#	Clinic BP Omron 715IT (Omron Corp., Tokyo, Japan)	CHX-MW 0.2%	66.2 (8.0)♦	68.0 (6.1)♦	2.2 (4.3)♦
II Sundqvist et al. (2016)	Omron M10-IT	CHX-MW 0.2%	NA♦	67.4 (4.5)♦	NA♦
		Placebo	NA♦	67.1 (4.5)♦	NA♦
III Tribble et al. (2019)#	Omron 10	CHX-MW 0.12%	78.1 (7.3)♦	78.4 (8.5)♦	0.3 (?)◊
IV Bescos et al. (2020)^	Connex ProBP 3400 Digital Blood Pressure Device, Welch Allyn UK Ltd.	CHX-MW 0.2%	62.8 (6.7)♦	62.8 (5.9)♦	0.0 (3.5)♦
		Placebo	NA♦	62.8 (6.7)♦	NA♦
V Bescos et al. (2024)	Connex ProBP 3400 Blood Pressure Device, Welch Allyn UK Ltd.	CHX-MW 0.2%	65.7 (7.2)♦	63.5 (6.2)♦	-2.2 (6.4)♦
		Control	61.3 (4.5)♦	60.2 (5.5)♦	-1.1 (4.1)♦

? Unknown/not provided
◊ Calculated by the authors of this review based on the data presented in the selected paper
♦ Provided by the original author
NA Not applicable
\# No control group (only a CHX-MW group); participants served as their own controls.
^ Observational crossover design; end scores after placebo use served as baseline for the CHX measurement, and participants served as their own controls.

Online Appendix 4. Overview of the included studies and the methodological items applied in the three types of meta-analysis performed.

		Control group		Meta-analysis
**Study, authors (year)**	**Research design**	**Control group**	**Subjects were own controls**	**Baseline–end of the CHX group #figure 2**
(I) Kapil et al. (2013)	observational	no	yes	yes
(II) Sundqvist et al. (2016)	RCT	yes	NA	no
(III) Tribble et al. (2019)	observational	no	yes	yes
(IV) Bescos et al. (2020)	observational	no	yes	yes
(V) Bescos et al. (2024)	RCT	yes	NA	yes

Online Appendix 5 – List of abbreviations

Abbreviation	Meaning
ABPM	Ambulatory blood pressure measurements
BP	Blood pressure
BVS	Bregje van Swaaij
CCT	Controlled clinical trial
CHX	Chlorhexidine
CI	Confidence interval
CVD	Cardiovascular disease
DBP	Diastolic blood pressure
DES	Dagmar Else Slot
DiffM	Difference of means
LSJT	Lars Sandor Jozephus Toonen, first author of this paper
MA	Meta-analysis
MLV	Melissa Leentje Vagevuur
MW	Mouthwash
NA	Not applicable
NO	Nitric oxide
NO_3	Nitrate
NR	Not reported
RCT	Randomized controlled trial
RevMan	Review Manager
SBP	Systolic blood pressure
SD	Standard deviation
SR	Systematic review
